# The Neural Pathway of Reflex Regulation of Electroacupuncture at Orofacial Acupoints on Gastric Functions in Rats

**DOI:** 10.1155/2012/753264

**Published:** 2012-12-31

**Authors:** Jianhua Liu, Wenbin Fu, Wei Yi, Zhenhua Xu, Nenggui Xu

**Affiliations:** ^1^Laboratory of Electrophysiology, The Secondary Medical College, Guangzhou University of Traditional Chinese Medicine, Guangzhou 510120, China; ^2^Key Laboratory of Acupuncture of Guangdong Province, Guangzhou University of Traditional Chinese Medicine, Guangzhou 510006, China

## Abstract

Acupuncture has a reflex regulation in gastrointestinal functions, which is characterized with segment. In the present study, the neural pathway of electroacupuncture (EA) at orofacial acupoints (ST2) on gastric myoelectric activity (GMA) in rats was investigated. The results indicated that EA at ST2 facilitated spike bursts of GMA, which is similar to EA at limbs and opposite to EA at abdomen. The excitatory effect was abolished by the transaction of infraorbital nerves, dorsal vagal complex lesion, and vagotomy, respectively. In addition, microinjection of L-glutamate into the nucleus of the solitary tract (NTS) attenuated the excitatory effect. All these data suggest that the dorsal vagal complex is involved in the reflex regulation of EA at orofacial acupoints on gastric functions and NTS-dorsal motor nucleus of the vagus (DMV) inhibitory connections may be essential for it.

## 1. Introduction

Acupuncture has been applied in the clinic to treat gastrointestinal (GI) diseases for thousands of years in China and Zusanli acupoint (ST36) is viewed as a classic acupoint in treating GI diseases in the textbook of Traditional Chinese Medicine. Recently, numerous clinical and experimental studies have shown that acupuncture is effective in treating GI disorders and regulating GI functions, including gastric motility, gastric acid secretion, and gastric myoelectric activity (GMA) [1–7]. It is noteworthy that, however, acupuncture at different regions induces different effects. For example, acupuncture at limbs enhances GI motility via vagal efferents [2, 3, 7], and acupuncture at the abdomen inhibits GI motility via sympathetic efferents [4], suggesting that the reflex regulation of acupuncture on GI tract is characterized with segment. In these studies, investigators notice that superspinal structures are involved in this process and dorsal vagal complex (DVC) may be an important candidate [1, 2, 4]. In our previous works, electroacupuncture (EA) at ST36 or orofacial acupoints promote GMA and induce c-fos expression in the nucleus of the solitary tract (NTS) [2, 8, 9]. Meanwhile, electrophysiological data show that there are convergent neurons of somatoviscera in the NTS simultaneously reactive to acupuncture stimuli and gastric distension [10]. It seems that DVC may be a major target of acupuncture in the regulation of gastric functions.

The DVC consists of the NTS, which receives primary afferent input from the GI tract, and the dorsal motor nucleus of the vagus (DMV), which contains the efferent vagal motor neurons innervating the GI tract. Therefore, the DVC is considered as a parasympathetic preganglionic center in the regulation of GI functions. Anatomical and electrophysiological data demonstrate the existence of excitatory and inhibitory synaptic connections between the NTS and DMV. However, most studies are focused on the inhibitory connections, in which excitation of NST neurons produces inhibition of postsynaptic neurons in the DMV projecting to the GI tract and influences output of the vagus nerves [11–16]. Therefore, the connections, especially inhibitory connections, may play an important role in the regulation of GI functions. 

SiBai (ST2) is located on the stomach meridian, which is mainly used to treat eye and GI diseases [17]. YangBai (GB14), as a control, is located on the urinary bladder meridian, which is mainly applied to treat headache and eye diseases [17]. In the present study, reflex regulation of EA at orofacial acupoints (ST2 and GB14) on GMA in rats and its neural pathway are investigated to clarify the underlying mechanism of EA on GI tract.

## 2. Experimental Procedure

### 2.1. Experimental Design

The study was divided into five parts: (1) to observe the effect of EA at ST2, which is located in the infraorbital foreman, on GMA. At the meanwhile, GB14, which is located in the forehead and 2.5 cm directly above the pupil, were chosen as the control. (2) To investigate the afferent pathway of EA at ST2 on GMA by transaction of the infraorbital nerves (ION). (3) To study the role of DVC in the EA effect following lesion of the DVC. (4) To explore the effect of microinjection of L-glutamate into the NTS on EA effects. (5) To observe the efferent pathway of EA effects by vagotomy.

### 2.2. Animals

 Adult Sprague-Dawley rats of both sexes, weighing from 220 to 250 g, were used in this study. Each rat was housed in controlled environmental conditions (25 ± 1°C, relatively humidity 40–60%, a 12 h/12 h light-dark cycle from 7:00 am to 19:00 pm), with access to food and water ad libitum. The procedures were performed in accordance with guidelines of Guangzhou University of Traditional Chinese Medicine for Care and Use Committee of Research Animals.

### 2.3. Implantation of Gastric Electrodes

The surgical procedure was similar to what was previously reported [2]. After an overnight fast, the animal was anesthetized with urethane (1 g/kg, i.p.) and laparotomy was made to expose the stomach. One 2-mm-long ring platinum electrode was sutured to the serosal surface on the anterior wall of the gastric antrum, about 0.5 cm proximal to the pyloric sphincter, and the other about 1.5 cm distal to that. Wires connecting with electrodes were brought out on the scruff of the neck through a subcutaneous tunnel. Finally the abdominal cavity was closed. 

### 2.4. Recording of GMA

Recording of GMA was performed as previously described [2]. The experiment was initiated after the rats were given about 5 days to recover completely from the surgery. All rats were fasted for 24 hours before the experiment and anesthetized with urethane (1 g/kg, i.p.). The low and high cutoff frequency of the amplifier was 10 Hz and 30 Hz, respectively, and both slow waves and spike bursts superimposed in the slow waves were continuously recorded for at least 10 min.

### 2.5. Transaction of ION

ION pretreatment was performed five days before EA at ST2. The animals were anesthetized by an i.p. injection of urethane (1 g/kg.). A vertical incision was made in the skin overlying the infraorbital foramen to expose bilateral ION under a dissecting microscope. For the transaction of ION (*n* = 6), the exposed ION was ligated at two separate points with silk suture, and the nerve bundle between two ligatures was transected. For the sham operation (*n* = 6), the ION were only exposed and did not receive any treatment.

### 2.6. Lesion of the DVC

Rats (*n* = 6) were anaesthetized with urethane (1 g/kg, i.p.) and were mounted in the stereotaxic apparatus (SR-6N, Narishige, Japan) in the prone position. The atlanto-occipital membrane and cerebellum were removed to expose the dorsal medulla. Obex is defined as the point between the area postrema and calamus scriptorius, where the central canal starts to open into the fourth ventricle [18]. Using this as the reference point, the insulating tungsten electrode was inserted into the DVC and 2 mA cathode current was applied for 10 s. Coordinates of the DVC are 0.5–0.7 mm rostral to the obex, 0.5 mm lateral to the midline bilaterally and 0.4 mm dorsal to the brainstem surface [18]. For the sham lesion (*n* = 6), electrodes were inserted into the same location without current.

### 2.7. Microinjection of L-Glutamate into the NTS

Rats (*n* = 6) were anaesthetized with urethane and the dorsal medulla was exposed as described above. Before EA at ST2, L-Glutamate (5 nmol/50 nl) (Sigma, USA) was microinjected into the NTS (coordinates: 0.5–0.7 mm rostral to the obex, 0.5 mm lateral to the midline bilaterally and 0.3–0.4 mm dorsal to the brainstem surface). Normal saline was microinjected in the same location as a control (*n* = 6).

### 2.8. Vagotomy

Prior to EA stimulation, rats (*n* = 6) were anaesthetized with urethane (1 g/kg, i.p.) and pretreated with vagotomy. Bilateral vagus nerves (VN) around the esophagus near the cardia were carefully isolated from the surrounding tissues and ligated at two separate points with silk suture, and the nerve bundle between two ligatures was transected. For the sham operation (*n* = 6), VN were only exposed and did not receive any treatment.

### 2.9. EA Treatment

Rats were anaesthetized with urethane (1 g/kg, i.p.) and immobilized in a plastic box. Two stainless acupuncture needles (0.28 mm outer diameter) were subcutaneously inserted 5 mm into the ST2 or GB14 acupoints on each side, and were left for 20 min. The electrical stimulation was from a medical EA apparatus (Model G6805-2, Shanghai, China). The stimulation parameters were a frequency of 2 and 20 Hz, alternatively, and intensity strong enough to only elicit slight twitches of the orofacial areas. ST2 and GB14 are located in the infraorbital foramen and 2.5 cm directly above the pupil on the forehead, respectively [19].

### 2.10. Histology

At the end of microinjection, the microinjection site was marked by injecting 50 nl of 2% pontamine sky blue. The rats were then perfused through the ascending aorta, with 100 ml of normal saline followed by 400 ml of 4% paraformaldehyde. The brainstem was removed and post-fixed in the same fixative solution for 6–8 h and soaked overnight in 20% sucrose solution. 40 *μ*m frozen transverse sections were obtained at −20°C by a freezing microtome (CM1850, Leica, Germany). Finally, brainstem sections were stained with neutral red to determine placement of micropipette by microscope.

For the lesion of DVC, the rats were perfused and fixed as described above, and frozen transverse sections were treated by hematoxylin-eosin (HE) staining to identify the location of lesion under microscope.

### 2.11. Statistic Analysis

Data were presented as mean ± standard error of the mean (SEM) and the significance level was set at *P* < 0.05. The results were analyzed using the paired-samples or independent-samples *t* test. In case of abnormal distribution or heteroscedasticity, the results were treated by nonparametric tests (Mann-Whitney *U*).

## 3. Results

### 3.1. Effects of EA at Orofacial Acupoints on GMA

**Figure 1:**
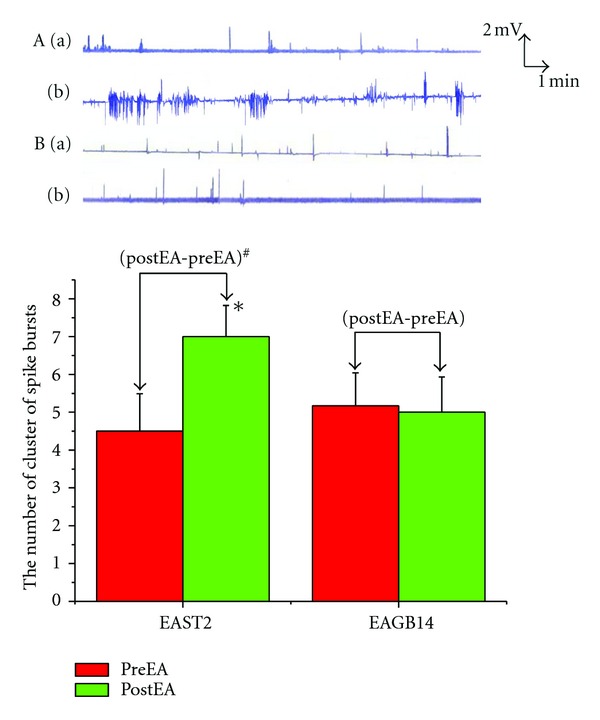
: Effects of EA at orofacial acupoints on GMA. *P < 0.05 versus preEAST2. ^#^ *P* < 0.05 versus (postEA-preEA)EAGB14. (A) EA at ST2 group; (B) EA at GB14 group. (a) PreEA; (b) postEA.

EA at ST2 produced a significant increase in the number of cluster of spike bursts per minute (4.50 ± 0.99  versus  7.00 ± 0.82, *P* < 0.05); however EA at GB14 did not (5.17 ± 0.87  versus  5.00 ± 0.93,  *P* > 0.05). Changes of spike bursts following EA at ST2 was significantly higher than that of EA at GB14 (1.33 ± 0.21  versus  0.17 ± 0.48, *P* < 0.05) (Figure 1).

### 3.2. Effects of ION Transaction on GMA Induced by EA

**Figure 2:**
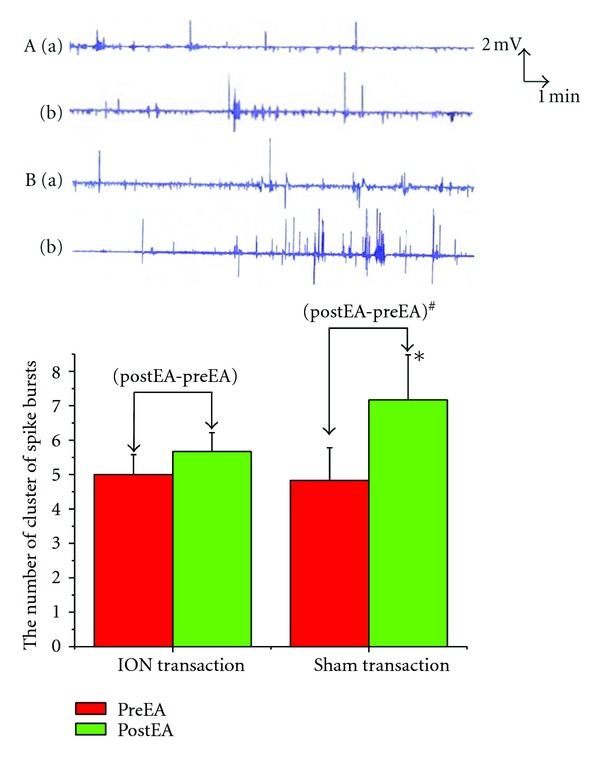
Effects of ION transaction on GMA induced by EA. *P < 0.05 versus preEA (sham transaction). ^#^ *P* < 0.05 versus (postEA-preEA) (ION transaction). (A) EA at ST2 and ION trancsaction; (B) EA at ST2 and sham transaction. (a) PreEA; (b) postEA.

After transaction of ION, EA at ST2 did not produce any change in the GMA (5.00 ± 0.58  versus  5.67 ± 0.55, *P* > 0.05). In the sham-operated group, EA at ST2 induced remarked increase in the spike bursts (4.83 ± 0.95  versus  7.17 ± 1.31, *P* < 0.05), which was similar to the change following EA at ST2 alone. Changes of the spike bursts following ION transaction was obviously lower than that in the sham operated group (0.67 ± 0.33  versus  2.33 ± 0.67, *P* < 0.05) (Figure 2). 

### 3.3. Effects of Lesion of DVC on GMA Induced by EA

**Figure 3:**
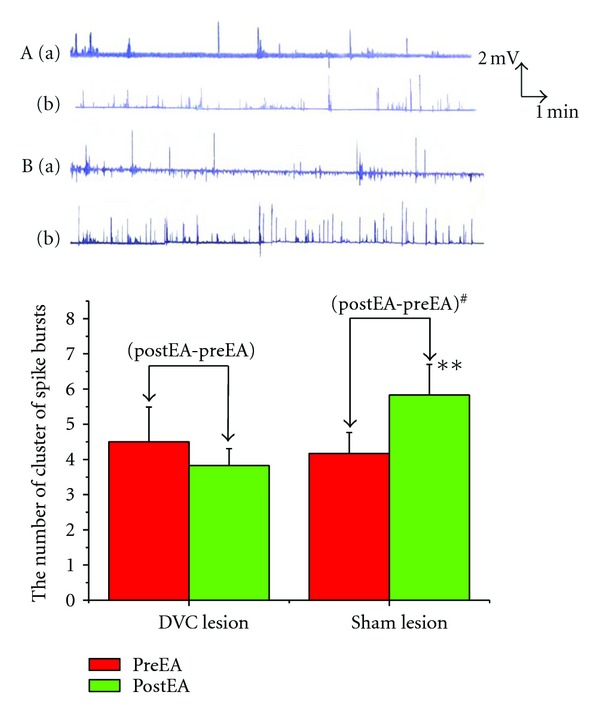
Effects of Lesion of DVC on GMA induced by EA. **P < 0.01 versus preEA (sham lesion). ^#^ *P* < 0.05 vesrus (postEA-preEA) (DVC lesion). (A) EA at ST2 and DVC lesion; (B) EA at ST2 and sham lesion. (a) PreEA; (b) postEA.

Following electrical lesion of the DVC, EA at ST2 had no any effect on the spike bursts (4.50 ± 0.99  versus  3.83 ± 0.48, *P* > 0.05). In the sham lesion group, EA at ST2 produced significant increase in the number of spike bursts (4.17 ± 0.60  versus  5.83 ± 0.87, *P* < 0.01). Changes of the spike bursts following lesion of DVC was obviously lower than that in the sham lesion group (0.67 ± 0.42  versus  1.67 ± 0.33, *P* < 0.05) (Figure 3).

### 3.4. Effects of Microinjection of Glutamate into the NTS on GMA Induced by EA

**Figure 4:**
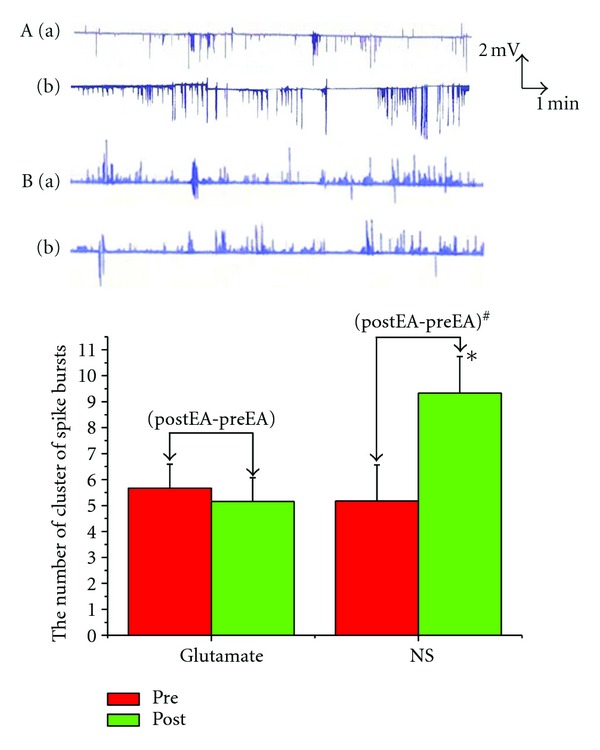
Effects of microinjection of glutamate into the NTS on GMA induced by EA. *P < 0.05 versus preEA (NS). ^#^ *P* < 0.05 versus (postEA-preEA) (Glutamate). (A) EA at ST2 and glutamate microinjection; (B) EA at ST2 and NS microinjection. (a) PreEA; (b) postEA.

Pretreatment of microinjection of L-glutamate into the NTS, EA at ST2 did not induce remarkable changes in the spike bursts (5.67 ± 0.92  versus  5.16 ± 0.91, *P* > 0.05), which lasted for about 5–10 min. Normal saline microinjection had no effects on changes in the spike bursts elicited by EA at ST2 (5.17 ± 1.40  versus  9.33 ± 1.41, *P* < 0.05). Histology showed that all microinjections were located within the NTS. Changes of the spike bursts following microinjection of glutamate into the NTS was obviously lower than that in the saline microinjection (0.50 ± 0.56  versus  1.83 ± 0.87, *P* < 0.05) (Figure 4).

### 3.5. Effects of Vagotomy on GMA Induced by EA

**Figure 5:**
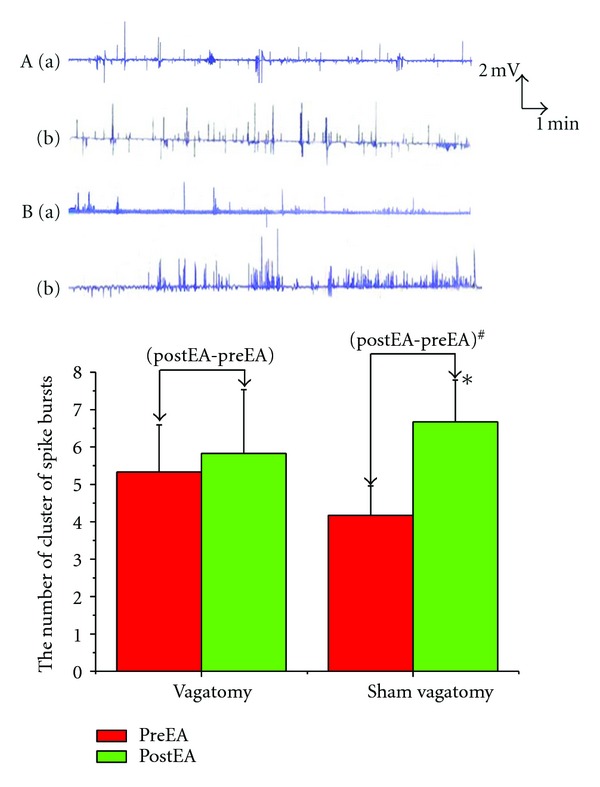
Effects of vagatomy on GMA induced by EA. *P < 0.05 versus preEA (sham vagatomy). ^#^ *P* < 0.05 versus (postEA-preEA) (vagatomy).

After bilateral vagotomy, EA at ST2 had no effect on the spike bursts (5.33 ± 1.26  versus  5.83 ± 1.70, *P* > 0.05). Sham operation did not influence the excitatory effects on GMA induced by EA at ST2 (4.17 ± 0.79  versus  6.67 ± 1.12, *P* < 0.01). Changes of the spike bursts following vagotomy was obviously lower than that in sham Changes of the spike bursts following microinjection of glutamate into the NTS was obviously lower than that in the saline microinjection operation (0.50 ± 0.67  versus  2.50 ± 0.5, *P* < 0.05) (Figure 5).

### 3.6. Histology

**Figure 6:**
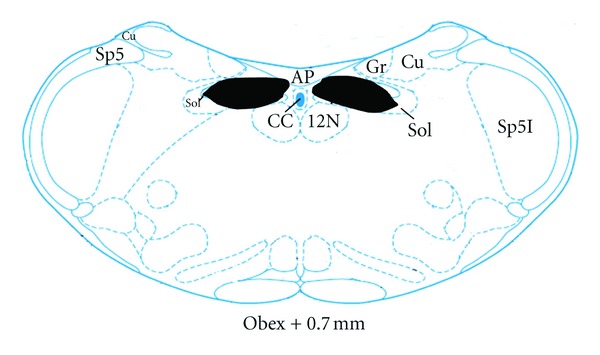
A diagrammatic representation of electrolytic lesion of DVC (shaded areas) (adapted from the atlas of Paxinos and Watson [18]). AP: area postrema; CC: central canal; Cu: cuneate fasciculus; Gr: gracile nucleus; Sol: nucleus solitary tract; sol: solitary tract; sp5: spinal trigeminal tract.

**Figure 7:**
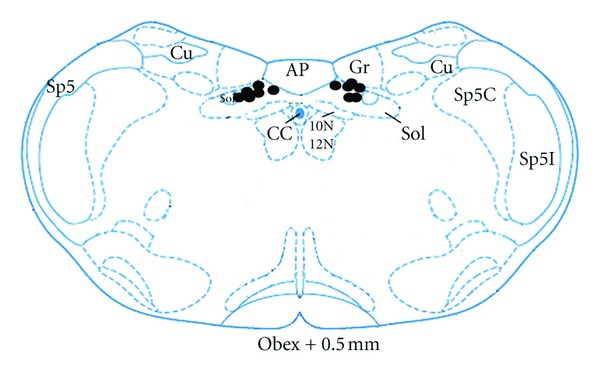
A diagrammatic representation of microinjection sites (*⚫*) within NTS (adapted from the atlas of Paxinos and Watson [18]). AP: area postrema; CC: central canal; Cu: cuneate fasciculus; Gr: gracile nucleus; Sol: nucleus solitary tract; sol: solitary tract; sp5: spinal trigeminal tract; Sp5C: spinal trigeminal nucleus, caudal part; Sp5I: spinal trigeminal nucleus, interpolar part; 10 N: dorsal motor nucleus of vagus; 12 N: hypoglossal nucleus.

A diagrammatic representation of electrolytic lesion of DVC and microinjection sites into the NTS is shown in Figures 6 and 7, respectively.

## 4. Discussion

It is well known that somatic inputs from skin and/or muscle induce changes in autonomic functions, which is called somato-autonomic reflex and characterized with segment. In anesthetized rats, pinching abdominal skin inhibits gastric motility and pinching limbs enhances gastric motility [20–22]. As one type of somatic stimuli, acupuncture has similar effects. Acupuncture at abdomen and lower chest inhibits gastric motility via sympathetic efferents, while acupuncture at limb facilitates the gastric motility via vagal efferents [4, 23]. Furthermore, EA at limb accelerates gastric emptying and enhance GMA in human and/or animals, which are also mediated by vagus nerve [6, 24]. In the present study, EA at orofacial acupoints (ST2) produce an increase in the number of cluster of spike bursts of GMA, which is abolished following bilateral vagotomy. GI motility is under the control of GMA, which is composed of slow waves (slow rhythmicity) and spikes (fast rhythmicity). The slow wave determines the frequency and propagation of gastrointestinal contractions and spike activities are superimposed on the slow waves and are electrical counterpart of contractions. GI contractions always occur when spikes are present. Therefore, EA at orofacial acupoints (ST2) produce excitatory effects on gastric motility and the vagus nerve is involved in the process, which is similar to EA at limbs and opposite to EA at abdomen, suggesting that both reflex regulation of acupuncture on gastric functions and the specific relationship between acupoints and viscera is characterized with segment.

In the study, the excitatory effect of EA at ST2 on the GMA is abolished by ION transaction, DVC lesion and vagotomy, respectively, suggesting that the neural pathway comprised by ION-DVC-VN is essential for this response. ST2 is located in the infraorbital foramen and innervated by infraorbital nerves, which have no direct projections to the DVC. Our previous works indicate that EA at ST2 induce c-fos expression in the NTS and inhibit visceral pain in rats, which is mediated by paratrigeminal nucleus and abolished by ION transaction [8, 9]. As a parasympathetic preganglionic center, DVC play an important role in the modulation of EA on GI functions. Anatomical evidence demonstrates that somatic afferents induced by EA at ST-36 is conveyed to the NTS and acts on the DMV, which promote gastric motility [23]. Moreover, NMDA receptors of gastric-projecting neurons in the DMV are involved in the regulation of EA at BL21 on gastric emptying in rats [25]. Our previous works also show that EA at ST36 enhanced the GMI and simultaneously inhibited release of Substance *P* in the DVC, which is abolished by transaction of vagus nerves [2]. 

Another interesting issue is that orofacial somatic inputs elicited by EA at ST2 act directly on the DMV neurons or indirectly on the DMV neurons via the NTS. Electrophysiological and anatomical data demonstrate the existence of substantial synaptic connections between the NTS and DMV and the inhibitory connections is a key for the modulation of GI functions [11–16]. It is well known that numerous neurotransmitters and neuromodulators in the DVC are involved in regulating of GI functions and a primary candidate is glutamate. In the vagovagal reflex, GI sensory inputs terminate in the NTS and release glutamate, which mainly act on non-NMDA receptors and activate the inhibitory neurons in the NTS projecting to the DMV and finally inhibit DMV neurons [26–28]. Several groups of investigators have shown that microinjection of glutamate into the DVC produces inhibitory or excitatory effect on gastric motility. It is seemed that glutamate microinjection into the NTS induces gastric inhibition [29, 30], whereas glutamate microinjection into the DMV results in gastric excitation [31, 32]. Electrophysiological data have shown that the activation of the paraventricular nucleus on gut-sensitive neurons in the DMV may be an indirect result of its effect on NTS neurons [33]. It has been demonstrated that NTS receives visceral and somatic sensory afferentsand plays an important role in the somato-visceral processing [2, 8–10]. Somatic inputs from EA at limbs act on DMV via NTS and promote gastric motility, and somatic inputs from EA at abdomen act on the rostral ventrolateral medulla (RVLM) via NTS and inhibit gastric motility [23]. In this study, microinjection of glutamate into the NTS inhibits the excitation of GMA induced by EA at ST2, suggesting that NTS-to-DMV inhibitory connections are involved in the regulation of EA at ST2 on parasympathetic motor output.

In conclusion, taken together, the above findings suggest that the neural pathway of ION-NTS-DMV-VN is involved in the reflex regulation of EA at orofacial acupoints on gastric functions and NTS-DMV inhibitory connections may be essential for it.
